# Ocular Findings in Patients with Orbital Fractures: A 1-Year Prospective Study in a Tertiary Center

**DOI:** 10.3390/medicina59061091

**Published:** 2023-06-05

**Authors:** Mohammed Asiri, Omir Aldowah

**Affiliations:** 1 Maxillofacial Department, Aseer Central Hospital, Ministry of Health, Abha 62523, Saudi Arabia; 2 Prosthetic Dental Science Department, Faculty of Dentistry, Najran University, Najran 11001, Saudi Arabia

**Keywords:** ocular, fracture, orbital

## Abstract

*Background and Objectives:* The aim of this study was to study the prevalence of ocular findings in patients with orbital fractures in a tertiary center in Saudi Arabia. *Materials and methods:* A cross-sectional observational study was performed. The participants were patients who presented with orbital trauma to the emergency department of King Saud Medical City (Riyadh, Saudi Arabia). Subjects included those diagnosed with isolated orbital fracture using clinical evaluation and CT examination. We performed direct evaluation of ocular findings for all patients. Variables studied included age, gender, site of ocular fracture, cause of trauma, side of fracture, and ocular findings. *Results:* In total, 74 patients with orbital fractures were included in this study (*n* = 74). Of the 74 patients, 69 (93.2%) were males and only 5 patients (6.8%) were females. The age range was 8-70 years, with a median age of 27 years. The 27.5–32.6-year age group was the most affected (95.0%). The left orbital bone was involved in the majority of bone fractures 48 (64.9%). The orbital floor (*n* = 52, 41.9%) and lateral wall (*n* = 31, 25.0%) were the most prevalent sites of bone fracture among the study patients. Road traffic accidents (RTAs) were the most common causes (64.9%) of orbital fractures, followed by assaults (16.2%) and then sports injuries and falls (9.5% and 8.1%, respectively). Animal attacks were the least cause of trauma (only 1 patient, 1.4%). The occurrence of ocular findings, either alone or in combination, showed that subconjunctival hemorrhage had the highest percentage (52.0%), followed by edema (17.6%) and ecchymosis (13.6%). A statistically significant correlation was reported between the site of bone fracture and orbital findings, with r = 0.251 * and *p* < 0.05. *Conclusions:* Subconjunctival bleeding, edema, and ecchymosis were the most frequent ocular abnormalities, in that order. There were a few instances of diplopia, exophthalmos, and paresthesia. Other ocular discoveries were incredibly uncommon. The location of bone fractures was found to be significantly correlated with ocular results.

## 1. Introduction

Facial trauma, including secondary orbital fractures, might be complicated by ocular injuries [1]. A comprehensive ocular examination of patients with trauma can greatly contribute to the understanding of the patients’ injury, recovery, and rehabilitation. Of all ocular injuries, 29% are related to orbital fractures [2]. 

Depending on the connections between the fracture site, visual acuity, the mechanism of injury, and even intraocular pressure, approximately one-third of patients presenting with an orbital fracture combined with moderate-to-severe ocular injury require intervention [3]. Ophthalmoplegia, paresthesia, exophthalmos, enophthalmos, diplopia, hyphema, reduced vision, vitreous hemorrhage, ruptured globe, edema, ecchymosis, emphysema, subconjunctival hemorrhage, lid laceration, ptosis, and restricted eye movement are the signs and symptoms that may be related to orbital trauma [4]. A CT scan is the gold standard for evaluating the condition of the orbital volume. For evaluating the bone walls of the internal orbit, it is significantly more sensitive than a clinical examination. One of the most common mistakes made when treating orbital fractures is failing to detect the full extent of the injury [5]. Sagittal cuts are also highly helpful when determining the condition of the orbital floor; however, coronal CT offers the finest image of the internal orbit. Axial slices are ideal for assessing the medial wall [6].

To provide patients with a high level of care, different treatment modalities should be considered by health providers. Severe injuries, such as ruptured globes, retinal tears, retrobulbar hemorrhages, and conjunctival lacerations, may require immediate intervention [3]. However, intervention is required within 2 days in the event of moderate damage, such as traumatic iritis, choroidal rupture, and lacerated eyelids. In contrast, subconjunctival hemorrhage and conjunctival ecchymosis are examples of mild injuries that do not require medical attention [3]. 

The location, age, and associated symptoms all affect the treatment options for orbital bone fractures. The degree of enophthalmos and diplopia determines how to explore and reconstruct blowout orbital fractures in the medial wall or floor. According to the majority of maxillofacial surgeons, between 50 and 75% of fractures necessitate exploration in order to release or realign the soft tissues of the orbit and reestablish the orbital volume [7].

Reports of incidents differ from one country to another and even within a country itself. This wide variation is influenced by various factors, most notably the specific mechanism of injury. In Riyadh, Saudi Arabia, road traffic accidents are associated with head and/or face injuries, which make up 30% of all injuries [8].

In addition, Alhammad et al. [9] reported that the fracture of the midface (64%), specifically the orbit (32%), is the most commonly fractured area. Although risk factors, such as motor vehicle accidents, have been well documented regionally [9,10,11], the prevalence of complications and findings of ocular and orbital fractures is scarce in Saudi Arabia. At the national level, Saudi Arabia’s literature on this subject is lacking, and thus, care given to those patients may suffer as a result of this scarcity. Furthermore, the data presented in this investigation may contribute to the creation of a database for significant orofacial fractures. Additionally, information from this kind of study may be used to encourage public awareness and well-being.

This study’s objective was to assess the prevalence of ocular findings in patients with orbital fractures. Additionally, it aimed to evaluate the correlation between ocular findings, the location of the orbital bone fracture, and the trauma’s causes.

## 2. Materials and Methods

The study was approved by the Institutional Review Board of Riyadh Elm University and registered with the number FPGRP/43736002/276 on 8 November 2018.

This study was implemented as a prospective cross-sectional observational study. The study population included all patients with orbital fractures seen in a tertiary center, King Saud Medical City, Riyadh, Saudi Arabia. The author (M.A.), with the approval of the included patients, conducted direct evaluation at the emergency department for a full year beginning in January 2019.

Using a custom data collection sheet, documentation for all patients with verified orbital fractures and ocular findings following evaluation by the maxillofacial surgery team and ophthalmologist at King Saud Medical City, Riyadh, was performed. Age, gender, the nature of the injury, and the location of the associated orbital fractures were recorded for each patient. The fractures were verified using computed tomography (CT) scans.

Exclusion criteria were as follows:

- Patients with orbital fractures associated with head, temporal, and/or brain trauma
- Patients with previous ophthalmic surgery or neurosurgery
- Patients on chronic medications, including steroids
- Patients with known chronic diseases, such as diabetes, hypertension, or neurological disorders
- Trauma under the influence of alcohol

Demographic data on age and gender were reported. In addition, the causes of injuries were classified into road traffic accidents (RTAs), falls, assaults, sports injuries, and animal attacks. All sites of orbital fractures were included, whether isolated or combined. They were classified into fractures of the floor, medial wall, lateral wall, roof, and/or rim.

Ocular findings included ruptured globes, subconjunctival hemorrhage, periorbital ecchymosis, chemosis, emphysema, diplopia, enophthalmos, exophthalmos, hyphema, vitreous hemorrhage, edema, retinal hemorrhage, reduced visual acuity, corneal laceration, lid laceration, muscle trapping, retinal detachment, paresthesia, and traumatic mydriasis.

To assess these parameters, a detailed history was taken, followed by computed tomography scanning of the brain and orbit.

A detailed ophthalmic assessment was performed using the diagnostic investigations listed in Table 1.

The data were analyzed using IBM^®^ SPSS Statistics version 22 (Chicago, IL, USA). Descriptive statistics were produced for all the variables of the study (frequency, percentage, mean, median, and standard deviation for the numeric variable, i.e., age). Inferential statistics (Spearman’s rho correlation) were used to find the correlation between pairs of variables. The significance level was set at *p* ≤ 0.05.

## 3. Results

In total, 74 patients with orbital fractures were included in this study (*n* = 74). Demographic data are presented in Table 2.

The left orbital bone was involved in the majority of bone fractures (*n* = 48, 64.9%), while the right orbital bone was engaged in 26 (35.1%) bone fractures.

Regarding the site of fracture, Table 3 represents the incidences of fracture sites.

**Figure 1:**
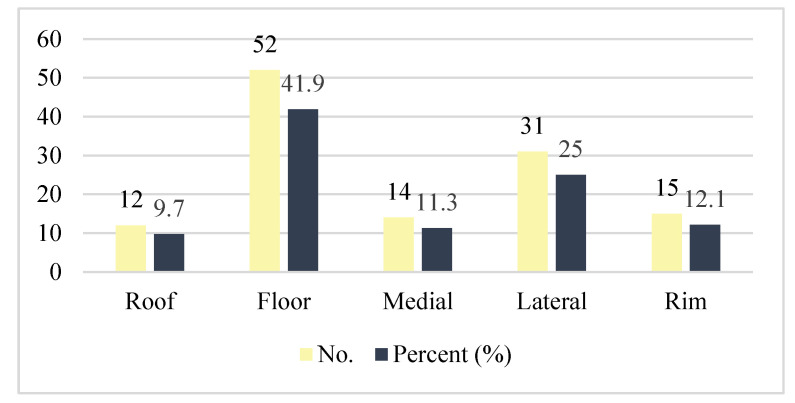
Sites of bone fracture.

**Figure 2:**
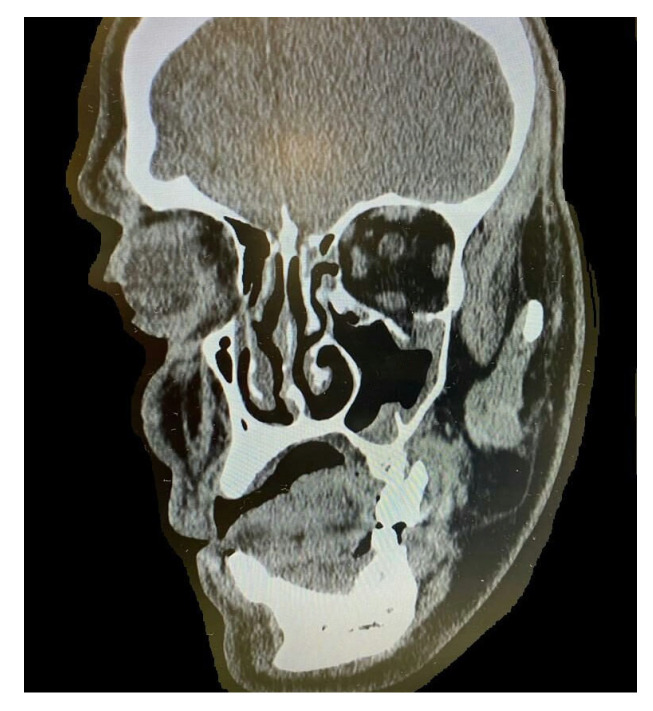
Cone-beam computed tomography (CBCT) of orbital floor fracture.

**Figure 3:**
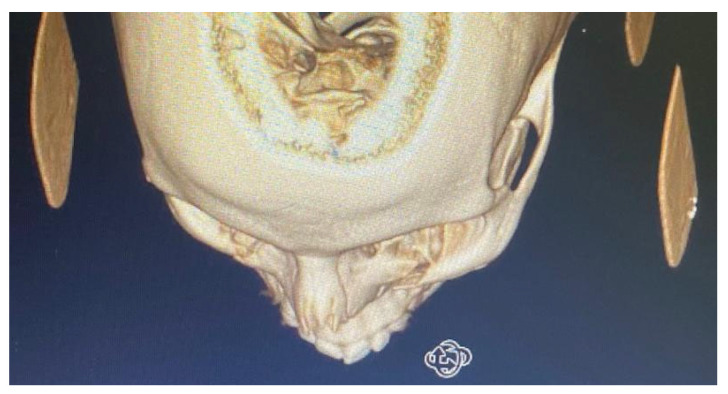
CBCT of left infraorbital rim fracture.

**Figure 4:**
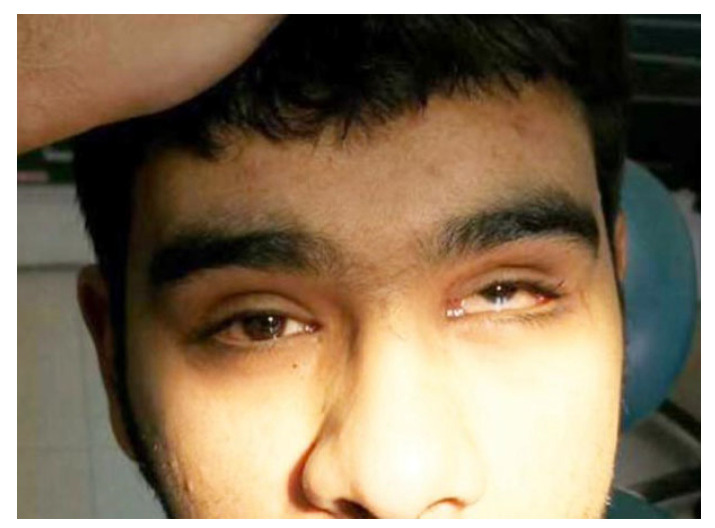
Orbital floor fracture with entrapment of the inferior rectus muscle; patients present with vertical gaze diplopia and restriction of upgaze.

**Figure 5:**
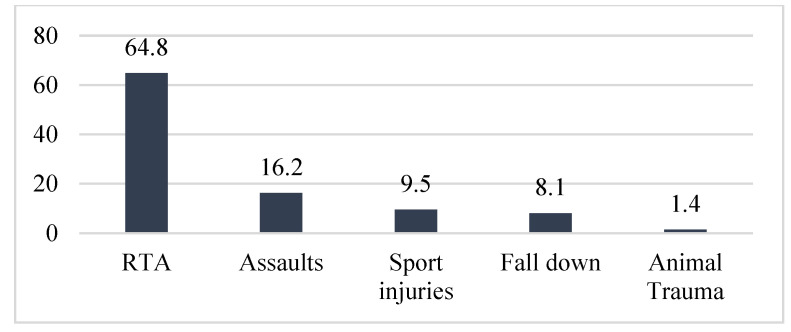
Causes of trauma.

By counting the occurrence of the sites of fracture, either alone or combined, we found that the orbital floor (*n* = 52, 41.9%; Figure 1) and lateral wall (*n* = 31, 25.0%) were the most prevalent sites of bone fracture among the study patients (Figure 2, Figure 3 and Figure 4). Figure 5 illustrates the distribution of the causes of trauma. 

**Figure 6:**
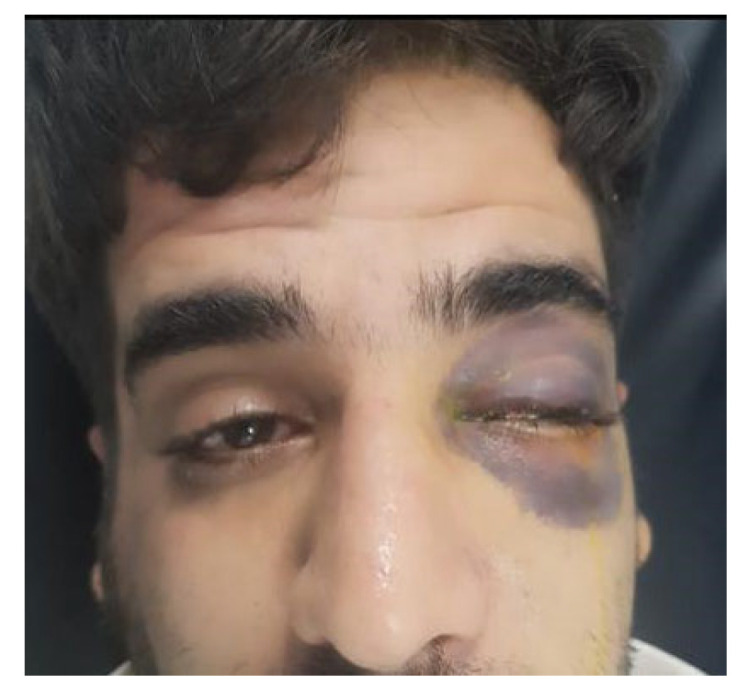
Entrapment of infraorbital muscle with ecchymosis, left eye.

The occurrence of ocular findings, either alone or in combination, showed that subconjunctival hemorrhage had the highest percentage (52.0%), followed by edema (17.6%) and ecchymosis (13.6%); see Figure 6. A detailed distribution is shown in Table 4.

**Figure 7:**
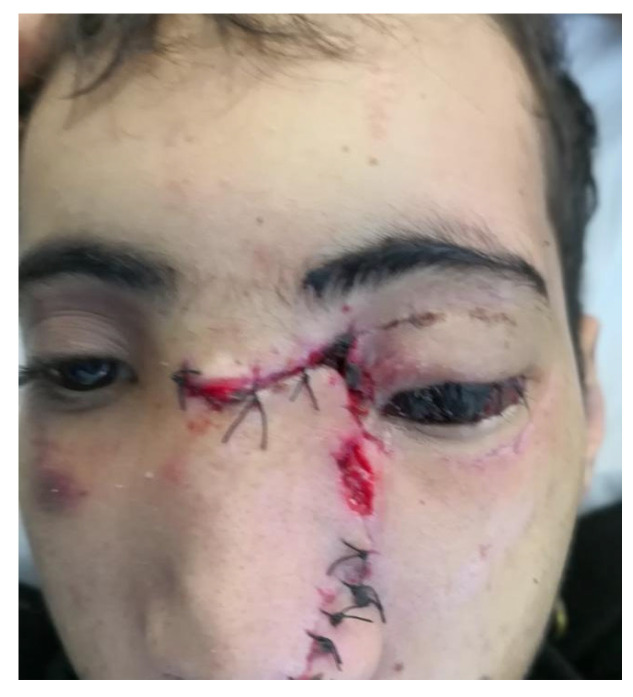
Ruptured globe, left eye.

With regard to patients with a single (isolated) ocular finding, the most prevalent finding was isolated subconjunctival hemorrhage (*n* = 30, 40.5%). Just three (4.1%) patients had isolated edema, and two (2.7%) presented with isolated ecchymosis. Similarly, there were two (2.7%) patients with only a ruptured globe (Figure 7), and only one (1.4%) patient was found to have just hyphema. However, there were 36 (48.6%) patients with more than one ocular finding, and the highest percentages were for subconjunctival hemorrhage and edema (10.8%) and subconjunctival hemorrhage and ecchymosis (9.5%); see Table 5.

Furthermore, Table 6 illustrates the prevalence of ocular findings per cause of trauma.

**Figure 8:**
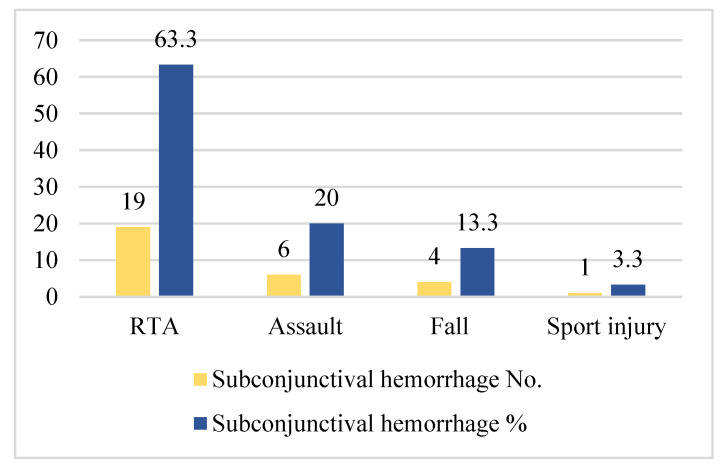
Cause of trauma with regard to subconjunctival hemorrhage.

To explore the association between ocular findings and the site of fracture and the cause of trauma, Spearman’s rho correlation coefficient was used. Ocular findings were associated with the site of bone fracture (*p* = 0.002), while the cause of trauma did not show statistical significance (Figure 8).

Table 7 illustrates Spearman’s correlation matrix among all the variables. A statistically significant correlation was reported between the site of bone fracture and orbital findings, with r = 0.251 * and *p* < 0.05. However, among all the study variables, no statistically significant correlation was reported, with *p* > 0.05. 

## 4. Discussion

The aim of this study was to report the prevalence of ocular findings in patients with orbital fractures in a tertiary center in Saudi Arabia. In the study, 74 patients with confirmed orbital fractures were evaluated. Most of the patients were males (93.2%), which reflected the Saudi Arabian rules regarding female driving licenses. Females have recently been allowed to drive.

The mean age of the patients was 30 years, and they were mainly injured due to RTAs (65%). Since this age group is recognized as a phase of great social excitement, careless driving on the road, and exposure to violence, the demand for ocular examinations has increased as well. The causes of ocular involvement in orbital fractures coincided with several other studies from other countries, such as the United Kingdom, the United States, Pakistan, and India [12,13,14,15].

The majority of orbital bone fractures (65%) involved the left orbital bone, which is similar to research conducted in Rome, Italy, between 2008 and 2013, which found that assault is the leading cause of fractures [16], which is related to the fact that most people are right-handed. The investigations of Altenon et al. [17] found that assault victims exhibit a prevalence of left orbital fractures, which is also similar to the findings of AI-Qurainy et al. [12]. Since the main cause of fracture in this study was RTAs, the site of fracture might be attributable to the driver’s position in the car (left). Although it was not determined whether the participants in this study were passengers or drivers, the authors’ position to interpret and their immersion in society and the workplace led them to assume that the majority of participants were drivers based on ethnographic theory [18]. Another study, conducted in Libya, reported that 42% of patients had injuries to their right eye, 38% had injuries to their left eye, and 20.3% had both eyes damaged [19]. Libya has the same driving system as Saudi Arabia. The authors believe that an examination of RTA reports by law enforcement or authorized entities could shed light on this issue, which could be the subject of additional research.

We discovered that the lateral orbital wall and the orbital floor are the most frequently fractured areas. These results concur with those of research conducted in the United States [20,21]. “Hydraulic theory,” which was stated first by King [22], explains that the orbital trauma transmitted through the eye to the orbital floor causes a rise in the intraorbital pressure, which is transferred to the walls of the orbit, creating a force great enough to cause a fracture of thin, weak bone. This theory can explain why orbital blowout fractures are so common. “Buckling theory” contends that a direct posterior movement force on the orbital rim results in a compression fracture of the wall along the orbital floor [23]. Due to its enormous open area and lack of support, the floor is the most frequently injured of the four orbital walls and frequently fractures after blunt face and orbital trauma [15]. As for lateral wall fractures, the mechanism of impact force from RTAs, where the lateral wall is harmed most of the time on the cusp of protecting the eyeball, may explain the high prevalence of RTAs identified in this study [14].

In this research, subconjunctival bleeding, edema, and ecchymosis were the most frequent ocular findings in relation to orbital injuries. The majority of the patients exhibited various eye symptoms. The most frequent finding in patients with a single (isolated) ocular abnormality was an isolated subconjunctival hemorrhage. Only three (4.1%) and two (2.7%) individuals developed isolated edema and ecchymosis, respectively. Similar to a study conducted in Boston, USA [20], and a study conducted in India [24], there were two patients (2.7%) with only ruptured globes, and only one patient (1.4%) was found to have merely hyphema.

Subconjunctival hemorrhage, a bright hemorrhagic patch on the bulbar conjunctiva brought on by the rupture and bleeding of a superficial, tiny capillary in the conjunctiva, was often the most common finding in this study. The inferior rectus fascia in the lower lid and the levator superioris and superior rectus fascia in the upper eyelid are both covered by the conjunctiva, which is also joined to the deep layer of the tarsus. The capillaries in this conjunctiva are numerous, fragile, and extremely thin, making them susceptible to rupture from minor trauma. Reassurance and local cold compresses for 24 h are typically used to treat subconjunctival hemorrhage; it normally resolves on its own in 2–4 weeks [23].

Edema, which is considered an early finding in periorbital tissues and may be accompanied by ecchymosis or surgical emphysema, was the second-most frequent ocular finding in our study. This makes examination of the orbit and evaluation of underlying structures problematic [23].

The prevalence of ecchymosis was ranked third in this study. Subconjunctival bleeding, periorbital ecchymosis, and lid laceration were quite prevalent, and ecchymosis can be explained by the assumption that the anterior region of the eye is more frequently involved in orbital fractures than the posterior segment [13].

This study’s paranesthesia rate of 4% is similar to a Glasgow investigation of the morbidity of the infraorbital nerve following orbitozygomatic complex fractures, which found that paresthesia associated with blowout fractures is unusual and has a lower prevalence [25]. Our findings refute the notion that if there is infraorbital nerve dysfunction, there virtually certainly is an orbital floor fracture.

The exophthalmos finding was uncommon (2%). The primary factor for this is that the majority of injuries in this study involved the eye’s anterior portion more frequently. Edema, pressure from a fractured bone on the eyeball, and bleeding, particularly a retrobulbar hemorrhage, can all contribute to the protrusion of the eyeball. A retrobulbar hemorrhage is an accumulation of blood in the retrobulbar space, which can stretch the optic nerve, obstruct ocular perfusion, cause proptosis, restrict extraocular movement, cause vision loss, and result in an afferent pupillary deficit. Intervention is urgently required in this situation [23].

In addition, enophthalmos was rarely observed. It results from a change in the globe’s soft-tissue shape or location brought on by an enlargement of the orbital cavity as a result of inferomedial wall fractures, which are typically brought on by physical attack [26]. This explains why enophthalmos was uncommon in this study. Avulsion of the suspensory ligament, which results in a decrease in the ocular level, known as hypophthalmos, is the second potential cause of enophthalmos. The majority of enophthalmos are regarded as late clinical signs induced by posterior recession of the globe when the eye lowers by 1–4 mm in later days following trauma, which is another rationale for the study’s low number of enophthalmos results [27]. This late discovery, which could appear in a few days or a few weeks, was perhaps concealed by bruising and swelling during the initial stages of the injury. Enophthalmos may also be brought on by untreated or improperly treated wounds, which were not evaluated in this study. Similar to previous findings [23], closed globe injuries were generally more frequent than open globe injuries.

This study had the drawback of being conducted at a single location for a whole year, and only early-stage preoperative patients’ ocular findings were assessed. Although the time for patient recruitment was 1 year, the sample size was limited. This is one of the limitations of convenience sampling. However, it might serve as the foundation for more research and draw the clinicians’ attention required to preserve crucial ocular structures. Thus, these findings and outcomes could benefit attending surgeons in making better diagnoses and treatment decisions for patients with ocular damage.

## 5. Conclusions

The most frequent fracture pattern in this study was an orbital floor fracture, followed by lateral orbital bone fractures. The orbital floor and lateral wall bones were the most typical sites of orbital bone fractures.

Subconjunctival bleeding, edema, and ecchymosis were the most frequent ocular abnormalities, in that order. There were a few instances of diplopia, exophthalmos, and paresthesia. Other ocular discoveries were incredibly uncommon.

The location of the bone fracture was found to be significantly correlated with ocular results, suggesting that this location may be an important factor in predicting ocular findings.

## Figures and Tables

**Table 1:** Ophthalmic assessment.

Assessment	Investigation Test
Pupillary response	A flashlight
Vision	Snellen’s chart
Extraocular movements	Bedside visual field assessment using finger confrontation (broad H test) and visual field tests
Fundus examination	Direct ophthalmoscope
Anterior segment	Slit lamp
Posterior segment on the dilated eye	Direct and indirect ophthalmoscopy
Intraocular pressure	Goldmann applanation tonometer
Enophthalmos or exophthalmos	An exophthalmometer to measure the anteroposterior position of the eyes
Diplopia	Diplopia charting in patients complaining of diplopia or restricted movement

**Table 2:** Patients’ demographics.

Variables	(Mean ± SD)	Min.	Max.	Median
Age	30.1 *±* 11.1 years	8 years	70 years	27 years
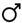	30.2 *±* 9.8 years	15 years	66 years	27 years
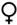	28.2 *±* 24.3 years	8 years	70 years	21 years
Gender	No.	Percentage (%)		
Male	69	93.2		
Female	5	6.8		
Total	74	100.0		

**Table 3:** Site of bone fracture per case.

Fracture Site	No.	Percentage
Roof	5	6.8
Floor	20	27.0
Medial wall	7	9.5
Lateral wall	4	5.4
Rim	2	2.7
Floor, lateral wall	15	20.3
Floor, rim, lateral wall	4	5.4
Rim, roof, floor	1	1.4
Floor, medial wall, lateral wall	4	5.4
Floor, roof, lateral wall	1	1.4
Roof, lateral wall	1	1.4
Roof, floor	1	1.4
Rim, roof	2	2.7
Floor, rim	4	5.4
Floor, rim, lateral wall, medial wall	2	2.7
Roof, medial wall	1	1.4
Total	74	100.0

**Table 4:** Prevalence of ocular findings.

Ocular Findings	No.	Percentage (%)
Subconjunctival hemorrhage	65	52
Edema	22	17.6
Ecchymosis	17	13.6
Paresthesia	5	4
Exophthalmos	3	2.4
Diplopia	3	2.4
Ruptured globe	2	1.6
Hyphema	1	0.8
Muscle trapping	1	0.8
Lid laceration	1	0.8
Emphysema	1	0.8
Corneal laceration	1	0.8
Reduced vision	1	0.8
Chemosis	1	0.8
Enophthalmos	1	0.8
Total	125	100

**Table 5:** Prevalence of ocular findings per case.

Ocular Findings	No.	(%)
Subconjunctival hemorrhage	30	40.5
Edema	3	4.1
Ecchymosis	2	2.7
Ruptured globe	2	2.7
Hyphema	1	1.4
Subconjunctival hemorrhage, edema	8	10.8
Subconjunctival hemorrhage, ecchymosis	7	9.5
Subconjunctival hemorrhage, edema, paresthesia	4	5.4
Subconjunctival hemorrhage, ecchymosis, edema	3	4.1
Subconjunctival hemorrhage, edema, exophthalmos	2	2.7
Subconjunctival hemorrhage, ecchymosis, paresthesia	1	1.4
Subconjunctival hemorrhage, diplopia, edema, muscle trapping	1	1.4
Subconjunctival hemorrhage, ecchymosis, diplopia	1	1.4
Subconjunctival hemorrhage, ecchymosis, exophthalmos	1	1.4
Subconjunctival hemorrhage, emphysema, ecchymosis, lid laceration	1	1.4
Edema, diplopia	1	1.4
Subconjunctival hemorrhage, edema, ecchymosis	1	1.4
Subconjunctival hemorrhage, corneal laceration	1	1.4
Subconjunctival hemorrhage, emphysema	1	1.4
Subconjunctival hemorrhage, reduced vision, chemosis	1	1.4
Subconjunctival hemorrhage, diplopia, enophthalmos	1	1.4
Subconjunctival hemorrhage, reduced vision	1	1.4
Total	74	100.0

**Table 6:** Prevalence of ocular findings by cause of trauma.

Ocular Finding		Cause of Trauma				
		RTA	Assault	Fall	Sports Injury	Animal Attack
Subconjunctival hemorrhage	No.	19	6	4	1	-
	%	39.6%	50.0%	66.7%	14.3%	-
Subconjunctival hemorrhage, edema	No.	4	3	1	0	-
	%	8.3%	25.0%	16.7%	0.0%	-
Subconjunctival hemorrhage, ecchymosis	No.	5	1	1	0	-
	%	10.4%	8.3%	16.7%	0.0%	-
Edema	No.	3	0	0	0	-
	%	6.2%	0.0%	0.0%	0.0%	-
Hyphema	No.	1	0	0	0	-
	%	2.1%	0.0%	0.0%	0.0%	-
Ecchymosis	No.	1	0	0	1	-
	%	2.1%	0.0%	0.0%	14.3%	-
Subconjunctival hemorrhage, ecchymosis, edema	No.	1	0	0	2	-
	%	2.1%	0.0%	0.0%	28.6%	-
Subconjunctival hemorrhage, edema, infraorbital nerve paresthesia	No.	1	2	0	1	-
	%	2.1%	16.7%	0.0%	14.3%	-
Subconjunctival hemorrhage, edema, exophthalmos	No.	2	0	0	0	-
	%	4.2%	0.0%	0.0%	0.0%	-
Subconjunctival hemorrhage, ecchymosis, paresthesia	No.	0	0	0	1	-
	%	0.0%	0.0%	0.0%	14.3%	-
Subconjunctival hemorrhage, diplopia, edema, muscle trapping,	No.	0	0	0	0	1
	%	0.0%	0.0%	0.0%	0.0%	100.0%
Subconjunctival hemorrhage, ecchymosis, diplopia	No.	0	0	0	1	-
	%	0.0%	0.0%	0.0%	14.3%	-
Subconjunctival hemorrhage, ecchymosis, exophthalmos	No.	1	0	0	0	-
	%	2.1%	0.0%	0.0%	0.0%	-
Subconjunctival hemorrhage, emphysema, ecchymosis, laceration lid	No.	1	0	0	0	-
	%	2.1%	0.0%	0.0%	0.0%	-
Edema, diplopia	No.	1	0	0	0	-
	%	2.1%	0.0%	0.0%	0.0%	-
Subconjunctival hemorrhage, edema, ecchymosis	No.	1	0	0	0	-
	%	2.1%	0.0%	0.0%	0.0%	-
Subconjunctival hemorrhage, corneal laceration	No.	1	0	0	0	-
	%	2.1%	0.0%	0.0%	0.0%	-
Subconjunctival hemorrhage, emphysema	No.	1	0	0	0	-
	%	2.1%	0.0%	0.0%	0.0%	-
Ruptured globe	No.	2	0	0	0	-
	%	4.2%	0.0%	0.0%	0.0%	-
Subconjunctival hemorrhage, reduced vision, chemosis	No.	1	0	0	0	-
	%	2.1%	0.0%	0.0%	0.0%	-
Subconjunctival hemorrhage, diplopia, enophthalmos	No.	1	0	0	0	-
	%	2.1%	0.0%	0.0%	0.0%	-
Subconjunctival hemorrhage, reduced vision	No.	1	0	0	0	-
	%	2.1%	0.0%	0.0%	0.0%	-

**Table 7:** Spearman’s rho correlation among variables.

Spearman’s Correlation Matrix						
			Orbital Finding	Cause of Trauma	Site of Fracture	Type of Bone Fracture
Spearman’s rho	Orbital finding	Correlation coefficient	1.000	0.005	0.019	0.251 *
		Sig. (2-tailed)	.	0.964	0.871	0.031
	Cause of trauma	Correlation coefficient	0.005	1.000	−0.093	0.106
		Sig. (2-tailed)	0.964	.	0.432	0.367
	Site of fracture	Correlation coefficient	0.019	−0.093	1.000	−0.030
		Sig. (2-tailed)	0.871	0.432	.	0.798
	Type of bone fracture	Correlation coefficient	0.251 *	0.106	−0.030	1.000
		Sig. (2-tailed)	0.031	0.367	0.798	.

* Correlation is significant at the 0.05 level (2-tailed).

## Data Availability

The data presented in this study are available on request from the corresponding author.
